# Aged Lymphatic Vessels and Mast Cells in Perilymphatic Tissues

**DOI:** 10.3390/ijms18050965

**Published:** 2017-05-03

**Authors:** Sarit Pal, Cynthia J. Meininger, Anatoliy A. Gashev

**Affiliations:** Department of Medical Physiology, College of Medicine, Texas A&M University Health Science Center, Temple, TX 76504, USA; spal@medicine.tamhsc.edu (S.P.); cjm@tamu.edu (C.J.M.)

**Keywords:** aging, lymphatic vessel, mast cell, histamine, NF-κB signaling

## Abstract

This review provides a comprehensive summary of research on aging-associated alterations in lymphatic vessels and mast cells in perilymphatic tissues. Aging alters structure (by increasing the size of zones with low muscle cell investiture), ultrastructure (through loss of the glycocalyx), and proteome composition with a concomitant increase in permeability of aged lymphatic vessels. The contractile function of aged lymphatic vessels is depleted with the abolished role of nitric oxide and an increased role of lymphatic-born histamine in flow-dependent regulation of lymphatic phasic contractions and tone. In addition, aging induces oxidative stress in lymphatic vessels and facilitates the spread of pathogens from these vessels into perilymphatic tissues. Aging causes the basal activation of perilymphatic mast cells, which, in turn, restricts recruitment/activation of immune cells in perilymphatic tissues. This aging-associated basal activation of mast cells limits proper functioning of the mast cell/histamine/NF-κB axis that is essential for the regulation of lymphatic vessel transport and barrier functions as well as for both the interaction and trafficking of immune cells near and within lymphatic collecting vessels. Cumulatively, these changes play important roles in the pathogenesis of alterations in inflammation and immunity associated with aging.

## 1. Introduction

Lymph flow is necessary for vital functions, such as fluid and macromolecule homeostasis, absorption of lipids and transport of immune cells. All of these functions require proper functioning of the lymphatic vessels (i.e., collecting lymphatics)—their phasic contractions that propel lymph forward to central veins, proper permeability and interaction with cellular elements of the surrounding tissue microenvironment [1,2,3,4,5,6,7,8]. Aging affects all of these functions of lymphatic vessels. However, despite findings of the last decades, our understanding of key regulatory mechanisms that support lymphatic vessel functions is still far from complete. The field of lymphatic biology has historically encountered difficulties in the assessment of lymphatic vessel function in vivo and in obtaining lymphatic vessels for studies in vitro. These difficulties have overlapped with an underappreciation of the importance of the lymphatic vascular component of the pathogenesis of various disorders in the past. Therefore, the lymphatic-related components in the pathogenesis of many diseases of the elderly remain to a large degree unknown.

Until the last decade, there were no published reports of systematic studies on aging-associated changes in the lymphatic vasculature. One study, published more than two decades ago, examined aging-associated changes in the structure of human lymphatic vessels [9]. These authors demonstrated that in older humans, the destruction of the elastic elements and atrophy of muscle cells in the thoracic duct wall resulted in the development of “duct sclerosis.” Investigations of the human mesenteric lymphatic bed demonstrated that after the age of 65, the number of collecting lymphatic vessels in the human mesentery was significantly reduced, and the number of connections between lymphatic vessels of the same lymphatic arcade level was greatly diminished. In some preparations of collecting lymphatic vessels, aneurism-like formations containing only endothelial cells in their walls were found, primarily in the areas located downstream but close to the lymphatic valves. Due to the profound difficulty of measuring lymph flow in vivo, there are only a few reports demonstrating measurements of reduced lymph flow in aged animals [10,11,12]. In particular, it was reported [12] that aging significantly reduced lymph flow from the main mesenteric lymph duct in rats by ~60% when compared between 3-month-old and 22-month-old animals.

Over the last decade, our group has performed a wide spectrum of studies significantly expanding our knowledge on how and by which mechanisms aging alters the structure and function of lymphatic vessels. These recent findings have led to a better understanding of the regulatory mechanisms of interactions between lymphatic vessels and mast cells (MCs) located in perilymphatic tissues, and demonstrated their importance for the control of all lymphatic functions mentioned above. We believe that these new discoveries provide the groundwork for a better understanding of the pathogenesis of many diseases in the elderly that involve a lymphatic component.

## 2. Aging-Induced Alterations of Lymphatic Muscle Cell Investiture and Their Potential Consequences for Lymphatic Vessel Function

Recently we performed quantitative evaluation of the aging-associated changes in muscle cell investiture in mesenteric lymphatic vessels (MLVs) isolated from rats of three age groups (3 months old: representing adolescence; 9 months old: representing adulthood, and 24 months old: representing elderly) [13]. Using immunohistochemical labeling of smooth muscle actin in the lymphatic vessel wall and subsequent quantitative analysis of obtained images of whole MLVs, we found specific aging-associated changes in the vessel structure. This analysis was performed with reference to the position of lymphatic valves and demonstrated unique features in the distribution of lymphatic muscle cells in valvular zones of lymphatic vessels. We determined that circularly oriented muscle cells located in post-valve zones of the lymphatic vessels constitute 92–95% of the total vessel length. Surprisingly, we did not find any significant aging-associated differences in the investiture of muscle cells in these post-valve zones. However, we demonstrated the existence of low muscle cell investiture zones predominantly located upstream (pre-valve zone) and above (valve zone) the lymphatic valves. These low muscle cell investiture zones in the lymphatic vessel wall consist predominantly of longitudinally oriented muscle cells that connect the pre-valve zone of the previous upstream lymphangion to the post-valve zone of the next downstream lymphangion. We demonstrated that these zones increased in size in aged animals, with aged MLVs losing their muscle cells predominantly around lymphatic valves. We believe that the thin-walled low muscle cell investiture zones in aged MLVs may, over time, be transformed into aneurysm-like formations, which were called “varicose bulges” in earlier studies mentioned above [9]. Virtually all aged MLVs possess zones in their walls that consist of only the thin endothelial cell layer in these abnormal formations. We proposed that the presence of such zones predisposes the aged lymphatic vessels to increased permeability, especially during inflammation. Given the fact that post-valvular zones in lymphatic vessels are ideal places for the formation of low-velocity turbulent lymph flow [14], these zones may result in a higher accumulation of various molecules, pathogens, and cancer cells here. Therefore, in aged lymphatic vessels, the higher lymphatic vessel permeability can facilitate the spread of foreign substances out of lymphatic vessels towards perilymphatic tissues. In addition, in the elderly, the deterioration of muscle cells surrounding lymphatic valves may limit the ability of aged lymphatic vessels to adequately adapt their contractility to various preload/afterload challenges through disrupted mechanisms of the lymphatic valve gating [15]. This may predispose the MLVs to lower lymphatic contractile/pumping productivity and cause an increase in the fraction of reversed flow [16] in aged lymphatic vessels. The latter might lead to lymph stasis and the potential spread of pathogens and cancer cells in a direction opposite to the direction of the normal lymph flow. Indeed, further studies performed by us, together with a collaborating laboratory, provided additional data to support these conclusions.

## 3. Aging-Induced Alterations of the Lymphatic Vessel Ultrastructure, Proteome Composition and Concomitant Increase in the Permeability of Aged Lymphatic Vessels

The study by Zolla et al. [17] provided a detailed analysis on how aging alters lymphatic vessel ultrastructure and its biochemical components. A comparative analysis of adventitia or the external surface of 9-month-old and 24-month-old rat MLVs by electron microscopy demonstrated signs of tissue degeneration with a substantial loss of extracellular matrix, specifically in the valve area region of these lymphatic collecting vessels. This correlated with findings of lower muscle cell investiture around lymphatic valves in aged MLVs [13], as discussed above. Furthermore, global proteomic profiling of lymphatic vessels showed that contractile and regulatory proteins like troponin; myosin cytoskeletal associated proteins like actin, dynein, and myosin binding proteins; extracellular proteins; and finally, several proteins associated with muscle contraction and generation of an action potential are significantly downregulated in aged lymphatic vessels. Such aging-associated changes in proteome composition may have their impact by altering contractile activity and lymph flow in aged lymphatic vessels. 

Other important findings were related to the aging-associated changes in the subglycocalyx space [17]. A significant loss of the glycocalyx, with reduction in size and continuity, was observed in lymphatic endothelial cells obtained from 24-month-old rats. An evaluation of proteomic differences among glycocalyx proteins in 9-month-old and 24-month-old rat lymphatic vessels revealed the following aging-associated changes. Cadherins, protocadherins, integrins, and gap junction proteins all showed a significant decrease (at least 2-fold) in MLVs harvested from aged as compared to adult rats. Additionally, complementing the ultrastructural analysis on the glycocalyx, proteomic analysis demonstrated several glycocalyx-associated proteins, including versicans, aggrecans, proteoglycans, brevican, galectins, mucins, agrin, and clusterins, were downregulated at least 2-fold in aged MLVs compared to their adult counterparts. 

Aging-associated changes in muscle cell investiture [13], together with alterations in glycocalyx composition and structure [17], affect the permeability of aged lymphatic vessels. We found that the permeability of aged lymphatic vessels increased in vivo, using Evans blue dye injected into the footpad of the hind limb of aged mice, as well as in ex vivo settings when fluorescently labeled bacteria were introduced into isolated, cannulated and pressurized aged rat MLVs [17]. A remarkable finding of these studies of isolated MLVs perfused with solutions containing fluorescently labeled *Staphylococcus aureus* was that in 9-month-old adult MLVs, bacteria had a tendency to accumulate near lymphatic valves (Figure 5k in [17]). These valvular zones, even in adult MLVs, were shown to have reduced muscle cell investiture in their walls [13]. In our opinion, this creates a predisposition for locally higher permeability of MLVs, and thus creates potential localized ports of exit for bacteria in aged MLVs. Therefore, in the study discussed above [17], significant fluorescence was observed in the surrounding solution due to escape of the fluorescently labeled bacteria from the lumen of aged MLVs (Figure 5l in [17]). Furthermore, experiments with the injection of various fluorescently labeled microorganisms (*Staphylococcus aureus*, *Cryptococcus neoformans*, and *Mycobacterium smegmatis*) into the footpad of 4-month-old and 22-month-old mice, demonstrated ~100 times more bacteria accumulated in the tissues surrounding the aged lymphatic vessels than the young ones [17]. These findings reinforce the observation that aging increases lymphatic vessel permeability, thus diminishing lymphatic vessel barrier function and compromising pathogen transport. 

## 4. Aging-Associated Alterations in Lymphatic Contractility and Lymph Flow

Over the last decade, we performed several in vivo as well as ex vivo studies where we characterized in detail how aging alters the contractile function of lymphatic vessels and, correspondingly, lymph flow. The results of these systematic studies provided important scientific information, which has not only a fundamental, but also a translational importance, as demonstrated below.

The first information on aging-associated alterations of the contractility and tone of lymphatic vessels was obtained in our studies with isolated segments of rat thoracic duct (TD) [18]. We found that the stretch-dependent regulation was altered in aged TD, especially at higher levels of pressure: negative inotropy, negative chronotropy and diminished minute pumping (2 to 3-fold decrease) were observed. Additionally, we found that the well-established role of nitric oxide (NO) in the imposed flow-dependent inhibition [19] was completely abolished in aged TD. At the same time, NO synthase (NOS) blockade by l-nitroarginine methyl ester increased pumping in a flow-independent manner. The potential explanation of these observations relates to the indications of the aging-associated depletion of endothelial NOS (eNOS) and an increase in inducible NOS (iNOS) in aged TD, which we believe is indicative of the presence of chronic aging-induced inflammation in TD. Western blot analyses demonstrated that the relative levels of eNOS were decreased ~7-fold in the 24-month-old TD when compared with 9-month-old TD; whereas iNOS levels were increased ~10-fold in 24-month-old TD [18].

Our studies with isolated segments of MLVs [20] revealed some similarities in aging-associated alterations of the contractility of these lymphatic collectors compared to TD. We found that aging remarkably weakens MLV contractility. The data also allowed us to propose the existence of another shear-dependent, but NO-independent, regulatory mechanism in the MLVs [20]. More recently [21], we established that the functional role of histamine as an endothelial-derived relaxing factor [22] is increased in aged MLVs, which occurs in parallel with the abolished role of NO in the reactions of these lymph vessels to increases in flow. In addition, we found an increased expression of histidine decarboxylase, the histamine-producing enzyme, in the endothelium of aged MLVs [21]. 

In another study, we evaluated the aging-associated changes in contractile characteristics of MLVs and in mesenteric lymph flow in vivo [23]. The active pumping of aged rat MLVs in vivo was found to be severely depleted, predominantly through an aging-associated decrease in lymphatic contractile frequency. An additional important finding of that study was that pumping in aged MLVs in vivo may be rapidly increased back to the levels of adult lymphatic vessels predominantly through the increase in contraction frequency induced by the experimental elimination of NO. These findings provided the first support for the existence of an additional, at that time unidentified, source of metabolites in aged perilymphatic mesenteric tissues that affects the aged MLVs. We also proposed that the aging-induced weakening of the mesenteric lymph pump might lead to a delay in the clearance of excessive fluid as well as potentially hazardous substances and foreign particles from the inflamed aged gut and mesentery [23]. Later studies provided additional support for this notion; the contractile reactivity of aged MLVs to acute inflammation in vivo was found to be abolished [24]. A recent study by another group also supported our data and the conclusions discussed above. The authors provided new evidence that the aging-associated loss of lymphatic tissue clearance correlated with a decreased lymphatic vessel density and a reduced lymphatic network complexity in the skin of aged mice as compared to younger controls [25].

## 5. Aging Alters the Functional Status of Activated Mast Cells in Perilymphatic Tissues

Paul Ehrlich first described MCs more than a century ago. Since then, our understanding of the function of MCs has largely been focused on their important contribution to allergic responses, anaphylaxis and infection [26,27,28]. There has been no in-depth consideration of the functional interactions between MCs and lymphatic vessels, with which MCs are commonly associated (MCs exhibit a 4.5-fold greater density near MLVs than in mesenteric tissue remote to MLVs) [29]. MCs can produce, store, and release, upon activation, numerous bio-active and, in particular, vasoactive mediators that continuously modulate the surrounding tissues [27,30,31,32,33,34,35,36,37,38,39]. In the past, some of these vasoactive mediators have been tested, via external delivery to lymphatic vessels, for their ability to influence lymphatic contractility. In particular, data from the literature suggest that histamine is a potent dose-dependent modulator of lymphatic contractility [40,41,42,43,44,45,46]. One study [45] directly linked pharmacologically-induced activation and degranulation of MCs to subsequent stimulatory changes in lymphatic contractility mediated by histamine, however, without reference to its concentration. Taking into account all of these data from the literature and our experimental data [23], we proposed that aging affects the functional status of perilymphatic MCs, which consequently influence the contractile activity of aged lymphatic vessels as well as other lymphatic functions.

Initially, we confirmed the presence of MCs in perilymphatic tissues by immunohistochemical labeling of proteins known to be present in MCs (mast cell tryptase, c-kit, prostaglandin D2 synthase, histidine decarboxylase, histamine, transmembrane protein 16A and tumor necrosis factor-alpha) [29]. Additionally, we verified results of the immunohistochemical labeling of MCs in the same segments of rat mesentery containing MLVs by labeling with Alexa Fluor 488-conjugated avidin followed by toluidine blue staining [29]. In that study, we confirmed a higher density of MCs located in close proximity to MLVs, and observed a 27% aging-associated increase in their total number in perilymphatic mesenteric tissues. In order to investigate the functional status of aged MCs located close to MLVs, we evaluated it by inducing MC activation with various agonists (substance P, anti-rat DNP immunoglobulin E, peptidoglycan from *Staphyloccus aureus* and compound 48/80) in the presence of Ruthenium Red, followed by staining with toluidine blue. We found a ~400% increase in the number of activated MCs in aged perilymphatic mesenteric tissues in resting conditions with a diminished ability to be newly activated when inflammatory or chemical stimuli were added acutely [29]. We concluded that the higher degree of pre-activation of MCs (with corresponding chronic aging-associated release of MC-derived mediators) in aged mesenteric tissue is highly likely to be responsible for the development of aging-associated impairments of contractile function of MLVs, as discussed above [23]. Furthermore, we hypothesized that the limited ability of aged MCs located close to the MLVs to react to the presence of acute stimuli may be considered as a contributory factor to the aging-associated deterioration in the immune response [29]. 

## 6. Aging Alters the Mast Cell-Directed Recruitment of Major Histocompatibility Complex (MHC) Class II Positive Cells and Eosinophils towards Mesenteric Lymphatic Vessels

In a follow-up study [47], we investigated the role of MCs in recruiting other immune cells towards MLVs and its aging-associated alterations. We treated live rat mesenteric tissue segments from rats of various ages for 2 h with MC activators (48/80 and substance P), and performed whole mount immunohistochemical labeling and vital dye staining of the mesenteric segments containing MLVs to identify immune cell recruitment towards MLVs after MC activation. The numbers of major histocompatibility complex (MHC) class II positive antigen presenting cells and eosinophils near MLVs were counted and compared between treatments and ages. Along with the previously mentioned greater density of MCs near MLVs, we demonstrated that mesenteric MC activation by compound 48/80 & substance P resulted in the recruitment of MHC class II positive cells and eosinophils towards MLVs. This effect was reduced in cromolyn-injected (MC-stabilized) rats, thus confirming that MCs are necessary for such recruitment. The immune cell presence near MLVs after MC activation was reduced in aged tissues [47]. We linked these findings [47] to our previous report [29] of lower numbers of intact MCs available for initiating an acute immune response in aged mesentery due to the aging-associated basal activation of MCs in perilymphatic tissues. Cumulatively, these findings served as an important step in investigations of the aging-associated alterations of the mechanisms that link MCs, lymphatic vessels and immune function.

## 7. Aging-Associated Oxidative Stress in Perilymphatic Tissues

It is well established that oxidative stress is an important factor contributing to vascular dysfunction with aging [48]. Vascular aging is associated with both structural and functional changes that can take place at the level of the endothelium, vascular smooth muscle cells and the extracellular matrix of vessels as well as in perivascular tissues, including MCs [49,50,51,52]. All of these changes were observed in aged lymphatic vessels and perilymphatic tissue compartments (as discussed above). It was also demonstrated that oxygen radicals significantly inhibited the contractility activity of rat MLVs [53]. To explore the aging-associated alterations in the status of the antioxidant regulatory systems in MLVs, we performed a study focused on the aging-induced changes in the expression of the major cellular antioxidant enzyme, superoxide dismutase (Cu/Zn-SOD, EC-SOD and Mn-SOD isoforms), peroxynitrite-mediated cellular damage and mitochondria-related superoxide radical production in aged MLVs [54]. We demonstrated the signs of aging-associated oxidative stress in the MLVs: increased superoxide, mitochondrial reactive oxygen species and thiobarbituric acid reactive substances. The decrease in total SOD enzymatic activity was linked to the predominant decrease in protein expression and immunohistochemical signal of the Cu/Zn-SOD protein isoform, as well as an increase of nitro-tyrosine in aged MLVs. The increased oxidative lipid and protein damage suggests that oxygen-derived radicals may be at least partially responsible for the aging-associated lymphatic pump dysfunction. In addition, we can consider aging-associated oxidative stress in perilymphatic tissues to be a likely cause of the basal activation of the aged perilymphatic MCs [29,47].

## 8. Aging Compromises the Mast Cell/Histamine/NF-κB-Mediated Reactions of Perilymphatic Mesenteric Tissues to Acute Inflammation

In another recent study, we established mechanistic links between aging-associated changes in the functional status of MCs and the altered responses of mesenteric tissues and MLVs to acute inflammation [24]. We used an in vivo model of acute peritoneal inflammation induced by lipopolysaccharide treatment of adult (9-month-old) and aged (24-month-old) rats. We analyzed the contractility of isolated MLVs, MC activation, and activation of nuclear factor-κB (NF-κB) without and with stabilization of MCs by cromolyn or a blockade of all types of histamine receptors in adult and aged perilymphatic mesenteric tissues. As mentioned above, we found that the reactivity of aged MLVs to lipopolysaccharide-induced acute inflammation was abolished. Importantly, in vivo studies demonstrated that the diminished reactivity of the mesenteric MCs located close to MLVs in aged animals to acute inflammation occurs due to the limited number of intact MCs available to be activated acutely in the aged mesentery [24]. These findings, obtained by two different experimental approaches using in vivo models of acute inflammation, complementing one another, provided strong support for the validity of our previous similar conclusions drawn from the acute ex vivo experiments discussed earlier [29,47]. Furthermore, we found that activated MCs trigger NF-κB signaling in the mesentery through the release of histamine. Moreover, in this study, we demonstrated that the pre-existing NF-κB activation is remarkably high in aged mesentery under resting conditions and there is no significant increase in NF-κB activation in aged mesenteric tissues located close to MLVs in response to acute inflammation. Therefore, the aging-associated basal activation of mesenteric MCs limits acute inflammatory NF-κB activation in aged mesentery and the lymphatic functions that follow, i.e., immune cell trafficking towards/through collecting lymphatic vessels and lymphatic permeability/barrier function.

Histamine itself has been historically known for its important role in immune cell functioning, including multi-directional crosstalk between MCs and macrophages, and chemoattraction of various immune cells by histamine [55,56,57,58,59]. At the same time, histamine also increases lymphatic permeability [60,61,62]. With respect to these roles of histamine, we believe that our data emphasize its crucial involvement in inflammatory events in lymphatic vessels and perilymphatic tissues.

As mentioned above, the functional status of MCs neighboring MLVs is important for the recruitment of MHC class II positive cells and eosinophils towards lymphatic vessels in cases of acute inflammatory stimulation, while aging alters this process, thereby decreasing the proper trafficking and activation of these immune cells in aged mesenteric perilymphatic tissues [47]. Our new data showing that MCs trigger the NF-κB-mediated reactions of mesentery to acute inflammation through histamine-involved regulatory mechanism(s) [24] allow us to assign a central role to the mast cell/histamine/NF-κB axis in lymphatic-related inflammatory events. In particular, we believe that novel data on lipopolysaccharide-induced modulation of neutrophil recruitment and macrophage polarization on lymphatic vessels [63] serve as convincing confirmation of our previous data discussed above [47] (macrophages, including CD11b positive cells, belong to MHC class II positive cells ([64] etc.)). In relation to the crucial role of the mast cell/histamine/NF-κB axis in this process and with consideration of the well-known role of NF-κB in the activation of CD11b positive cells [65], we believe that the smaller number of MCs available for acute inflammatory activation in elderly individuals will diminish NF-κB-driven CD11b cell activation. Since CD11b positive cells could be monocytes, macrophages, or even activated neutrophils [66], the aging-compromised mast cell/histamine/NF-κB activation alters the proper course of the innate immune response in aged mesentery. 

Another important process influenced by aging-associated alterations in the mast cell/histamine/NF-κB axis appears to be dendritic cell trafficking towards lymph nodes. This process is largely controlled by CCR-7 expression on dendritic cells and CCL21 secretion by the lymphatic endothelium [67,68,69,70], which has been demonstrated to be NF-κB/TNFα-dependent [71]. Furthermore, it has been demonstrated that PI3K/Akt1 and NF-κB signaling are important in CCR-7/CCL21-mediated survival and migration of dendritic cells [72]. In the healthy adult body, the mast cell/histamine/NF-κB axis is in fine balance between activated and non-activated states. This balance allows for the proper control of dendritic cell function. In aging, the normal balance in the mast cell/histamine/NF-κB axis is altered, so basal NF-κB signaling tends to be in a constitutively active state. As a result, the NF-κB-dependent increased basal reactivity of aged dendritic cells to self-antigens contributes to aging-associated chronic inflammation [73]. In turn, we believe that the reactivity of aged dendritic cells to an acute inflammation challenge will be compromised, thereby altering the proper course of events in the immune response to pathogens in the elderly population [24].

Alterations in immune cell trafficking towards/through collecting lymphatic vessels in the aged body co-exist with the aging-associated decrease in barrier function of lymphatic collectors, which creates a predisposition for an easier spread of pathogens towards perilymphatic tissues, as discussed above [17]. Recently, it has been demonstrated that lymphatic collecting vessel permeability is controlled by CCR7 in an IRF-4-dependent manner [74]. As IRF-4 expression is an NF-κB-dependent process [75], we believe that the mast cell/histamine/NF-κB axis is a major contributor to the maintenance of lymphatic permeability by mechanisms that involve the CCR-7/CCL21/IRF-4-dependent dendritic cells [74], and that aging-associated alterations in the function of this axis will affect lymphatic permeability and barrier function accordingly. Therefore, we conclude that proper functioning of the mast cell/histamine/NF-κB axis is necessary for reactions of the lymphatic vessels to acute inflammatory stimuli, as well as for the interaction and trafficking of immune cells near and within the collecting lymphatic vessels [24].

## 9. Conclusions

Studies during the last decade have demonstrated that aging alters the structure and contractile function of lymphatic vessels. These changes are complex and predispose aged lymphatic vessels to diminished lymphatic contractility and lymph flow, especially during edemagenic challenges in the event of overlapping acute inflammation in the elderly. In addition, aging creates conditions for the easier spread of pathogens from lymphatic vessels into perilymphatic tissues. Aging induces the basal activation of perilymphatic MCs, which, in turn, restricts the recruitment/activation of several types of immune cells in perilymphatic tissues. Activated MCs trigger NF-κB signaling through the release of histamine. The aging-associated basal activation of MCs limits acute histamine-driven inflammatory NF-κB activation in aged perilymphatic tissues. Therefore, aging-associated dysfunction of MCs critically affects all NF-κB-mediated reactions of aged tissues to acute inflammation. Proper functioning of the mast cell/histamine/NF-κB axis is essential for the regulation of lymphatic vessel transport and barrier functions, as well as for both the interaction and trafficking of immune cells near and within lymphatic collectors. Thus, this axis appears to play important roles in the pathogenesis of the alterations in inflammation and immunity associated with aging.

## Abbreviations

MLVs	Mesenteric lymphatic vessels
TD	Thoracic duct
MCs	Mast cells
NO	Nitric oxide
NOS	Nitric oxide synthase
MHC	Major histocompatibility complex
NF-κB	Nuclear factor-κB
SOD	Superoxide dismutase
